# Prevalence of anemia and associated factors among adult hypertensive patients in Referral Hospitals, Amhara Regional State

**DOI:** 10.1038/s41598-023-41553-z

**Published:** 2023-08-31

**Authors:** Yibeltal Yismaw Gela, Daniel Gashaneh Belay, Dagmawi Chilot, Amare Agmas Andualem, Desalegn Anmut Bitew, Deresse Sinamaw, Habitu Birhan Eshetu, Abdulwase Mohammed Seid, Wudneh Simegn, Anteneh Ayelign Kibret, Mohammed Abdu Seid, Mengistie Diress

**Affiliations:** 1 Department of Physiology, College of Medicine and Health Sciences, University of Gondar, Gondar, Ethiopia; 2 Department of Human Anatomy, College of Medicine and Health Sciences, University of Gondar, Gondar, Ethiopia; 3 Department of Anesthesia, College of Medicine and Health Science, Injibara University, Injibara, Ethiopia; 4 Department of Reproductive Health, College of Medicine and Health Sciences, University of Gondar, Gondar, Ethiopia; 5 Department of Epidemiology and Biostatics, Institute of Public Health, College of Medicine and Health Sciences, University of Gondar, Gondar, Ethiopia; 6 Center for Innovative Drug Development and Therapeutic Trials for Africa (CDT-Africa), Addis Ababa University, College of Health Sciences, Addis Ababa, Ethiopia; 7 Department of Biomedical Science, Debre Markos University, Debre Markos, Ethiopia; 8 Department of Health Promotion and Health Behavior, Institute of Public Health, College of Medicine and Health Sciences, University of Gondar, PO.Box.196, Gondar, Ethiopia; 9 Department of Clinical Pharmacy, School of Pharmacy, University of Gondar, Gondar, Ethiopia; 10 Department of Social and Administrative Pharmacy, School of Pharmacy, University of Gondar, Gondar, Ethiopia; 11 Unit of Human Physiology, Department of Biomedical Science, College of Health Sciences, Debre Tabor University, Debra Tabor, Ethiopia

**Keywords:** Medical research, Physiology, Cardiovascular biology, Diseases, Haematological diseases

## Abstract

Anemia is a risk factor for adverse cardiovascular disease outcomes in hypertensive patients. Chronic anemia increases preload, reduces afterload, and leads to increased cardiac output in hypertension patients. In the long term, this may result in maladaptive left ventricular hypertrophy, which in turn is a well-recognized risk factor for cardiovascular disease outcomes and all-cause mortality in hypertension. Low hemoglobin and hematocrit levels might be strongly indicate hypertensive end-organ damage, specifically kidney failure. Therefore, this study determined the prevalence of anemia and associated factors among hypertensive patients in Referral Hospitals, Amhara Regional State, Ethiopia, in 2020. An institution-based cross-sectional study was conducted in Amhara Regional Referral Hospitals from February 20 to April 30, 2020. Random and systemic sampling techniques were used to select 428 study participants. Data were entered and coded in to Epi data version 3.0 and then exported into STATA 14 for analysis. In bivariable logistic regression, variables with a p-value of < 0.25 were included in multivariable logistic regression. Using a 95% confidence interval, variables having a p-value ≤ 0.05 in multivariable logistic regression were declared as statistically significant variables. In this study, a total of 428 study participants were involved with 99.5% response rate. The prevalence of anemia among hypertensive patients was 17.6%, with a 95% CI (14.3–21.5%). Estimated glomerular filtration rate (eGFR) < 90 ml/min (AOR = 2.77, 95% CI 1.56- 4.92)], duration of hypertension (HTN) ≥ 5 years (AOR = 2.37, 95% CI 1.36–4.15), uncontrolled blood pressure (AOR = 1.91, 95% CI 1.08–3.35), and higher pulse pressure (AOR = 1.05 (95% CI 1.02–1.08) were significantly associated with anemia. Nearly one out of five hypertensive patients had anemia. Impaired estimated glomerular filtration rate, duration of HTN, blood pressure status, and pulse pressure were the independent predictors of anemia among hypertensive patients. Screening hemoglobin level at a regular interval is recommended for the hypertensive patients to take an appropriate intervention.

## Introduction

Hypertension is a systolic blood pressure of 140 mm Hg or greater and/or a diastolic pressure of 90 mm Hg or greater in subjects who are not taking antihypertensive medication^1^. Every year, raised blood pressure kills nine million people^2^. The prevalence of hypertension ranges from 17 to 40% in developed countries and almost two-thirds of patients have untreated and poorly controlled hypertension^3, 4^. A systematic meta-analysis in Ethiopia showed that the prevalence of hypertension was 19.6%^5^.

Generally, non-communicable diseases (NCDs) are the major cause of death in the world. In 2008, NCDs accounted for more than 36 million (63%) of the 57 million deaths^5^. High blood pressure (BP) is the major risk factor for cardiovascular diseases and chronic kidney disease, and a leading cause of mortality world-wide^6^.

There is endothelial dysfunction in hypertensive patients, which is manifested by increased levels of vascular cell adhesion molecule-1 (sVCAM-1), soluble intercellular adhesion molecule-1 (sICAM-1), and von Willebrand Factor (vWF) in mediating vessel injury in hypertension, which leads to high levels of circulating pro-inflammatory cytokines^7–9^.

Pro-inflammatory cytokines, such as tumor necrosis factor and interleukin-6, have direct inhibitory effects on the bone marrow, which may cause anemia in hypertensive patients^4, 10^. Hypertension also strongly affects the functional and physicochemical properties of red blood cells (RBCs). Studies have shown that circulating RBCs might be impaired in hypertensive patients due to alterations in sodium–lithium counter-transport and sodium–potassium ATPase activity of RBC membranes^11, 12^. RBC deformability decreases due to sodium–potassium ATPase activity of RBC membrane impairment^10, 12^.

Anemia is a risk factor for adverse cardiovascular disease outcomes in hypertension^13, 14^. Low hemoglobin levels are associated with high risk of coronary heart disease^13^. Chronic anemia increase preload, reduces afterload, and leads to increased cardiac output in hypertension patients^15^. In the long term, this may result in maladaptive left ventricular hypertrophy, which in turn is a well-recognized risk factor for cardiovascular disease outcomes and all-cause mortality in hypertension^15, 16^. Recently, low hemoglobin and hematocrit levels strongly indicate hypertensive end-organ damage, specifically kidney failure in hypertensive patients^17, 18^.

However, there is limited information concerning the prevalence of anemia among hypertensive patients in Ethiopia. Therefore, this study aimed to assess the prevalence of anemia and associated factors among hypertensive patients in Amhara Regional State.

## Methods and materials

### Study setting and period

An institution-based cross-sectional study was conducted in selected referral hospitals, Amhara Regional State from February 2020 to April 30, 2020.

### Study population

All adult hypertensive patients having follow-up at the University of Gondar Comprehensive Specialized and Felege Hiwot Referral Hospitals were included in the study.

All patients with diabetic mellitus, chronic kidney disease, human immunodeficiency virus infection, and hematological malignancies were excluded from the study.

### Sample size calculation and sampling procedure

The sample size was calculated using a single population proportion formula based on estimates of 50% anemia prevalence, 95% confidence interval, and a 5% marginal error.

$${\text{N}} = \left[ {\frac{{{ }\left( {{\text{Z}}_{{{\upalpha }/2}} } \right)^{2} \times {\text{p}}\left( {1 - {\text{p}}} \right){ }}}{{{\text{d}}^{2} }}} \right] = \left[ {\frac{{\left( {1.96} \right)^{2} \times 0.5\left( {1 - 0.5} \right){ }}}{{\left( {0.05} \right)^{2} }}} \right] = {385}$$

N: sample size, p: estimated prevalence value (50%), d: Margin of sampling error tolerated (5%), Z_α/2_ (1.96): critical value at 95% confidence interval of certainty.

After adding 11% of the non-response rate, a total of 428 participants were selected.

### Sampling procedures

There were five referral hospitals in Amhara Region during our data collection period. Of those, the University of Gondar Comprehensive Specialized and Felege Hiwot Referral Hospitals were selected using lottery methods of simple random sampling techniques.

**Figure 1:**
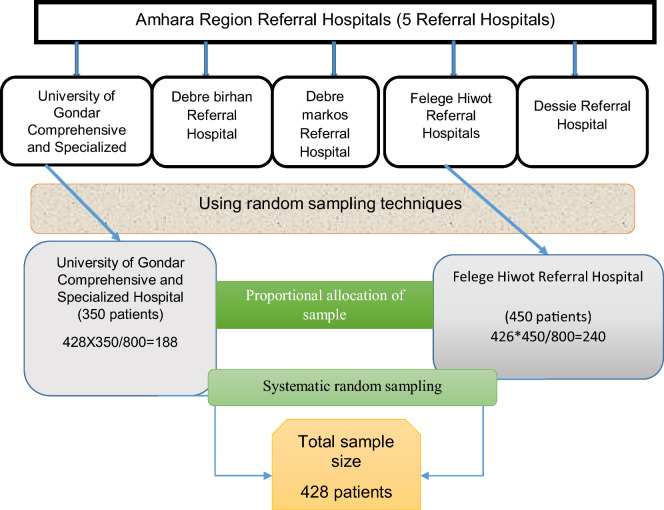
A flow chart describes the proportion of patients taking from the University of Gondar Comprehensive Specialized and Felege Hiwot Referral Hospitals, 2020.

Three hundred fifty and four hundred fifty hypertensive patients were encountered at the follow-up clinics of the University of Gondar Comprehensive Specialized and Felege Hiwot Referral Hospitals, respectively, at the time of the data collection period. Using a systematic random sampling technique, 188 and 240 HTN patients were selected from the University of Gondar Comprehensive Specialized and Felege Hiwot Referral Hospitals, respectively, with a K value of 2. The first patient was chosen by a lottery system, and then each of the first patients was interviewed (Fig. 1).

This research was carried out in accordance with the Helsinki Declaration's standards. The University of Gondar Institutional Review Board provided ethical clearance. Each study participant signed a written informed consent before the data collection began. For study participants who were unable to read or write, their parents or legally authorized representatives signed a written informed consent on their behalf. The privacy and confidentiality of study participant data were also carefully maintained.

### Operational definitions

Hypertension: a systolic blood pressure of 140 mmHg and/or a diastolic blood pressure of 90 mmHg (22) or who is taking antihypertensive medication^1^.

Anemia: a hemoglobin level less than 13 gm/dl in men and below 12 gm/dl in women for age ≥ 18 years old, as recommended by World Health Organization^19^.

Female participants with a blood hemoglobin level of (11–11.9 gm/dl), (8–10.9 gm/dl), and < 8 gm/dl have mild, moderate, and severe anemia respectively, whereas for male participants blood hemoglobin levels of (11–12.9 gm/dl), (8–10.9 gm/dl), and < 8 gm/dl have mild, moderate, and severe anemia, respectively^19^.

Impaired glomerular filtration rate: Patients who had an estimated glomerular filtration rate (eGFR) < 60 mL/min/1.73 m^2^ using EPI equations^20–22^.

Participants with a blood pressure of < 130/80 mmHg for at least three consecutive follow-up measurements were considered to have controlled blood pressure^23^.

Participants with a body mass index (BMI) of (< 18.5 kg/m^2^), (18.5–24.9 kg/m^2^), (25–29.9 kg/m^2^) where classified as underweight, normal, and overweight respectively, while BMI of ≥ 30 kg/m^2^ classified as obesity^24, 25^.

### Data collection procedures and tools

The data was collected using an interviewer-administered structured questionnaire that included socio-demographic information, medical card review, and weight and height assessment. Aside from the face-to-face interview, follow-up blood pressure records and patient cards were examined. The estimated glomerular filtration rate was calculated using the 2021 CKD-EPI creatinine equation, which is currently recommended for predicting GFR in adults 18 and older^26, 27^. The most recent creatinine and hemoglobin levels recorded in the medical record within one month prior to the time of data collection were taken.

A digital automatic blood pressure monitor was used to take blood pressure readings. Body weight and height were determined using a height measurement stand and a weighing machine. The participants' blood pressure, height, and weight were assessed according to established protocol, using World Health Organization protocol^28^.

### Data processing and analysis

After the data collected it were checked for its completeness, entered and coded in to Epi data version 3.0 and then exported to STATA 14 for analysis. Continuous variables were presented using mean and standard deviation, while for categorical variables like frequency and pie chart, were used.

The researchers used bivariable and multivariable logistic regression analyses. Variables associated with anemia with a p-value less than 0.25 using bi-variable logistic regression were included in the multivariable regression model. Finally, factors with a p-value of 0.05 or less in multivariable logistic regression were validated as significantly associated with anemia using a 95 percent confidence interval. The Hosmer and Lemeshow goodness test has been done to evaluate model fitness, and the value was 0.245.

### Data quality assurance

To assure the data quality, training was given by the principal investigator to the data collectors with regard to the questioners, and the methods of data extraction from the patients’ charts. Four BSc nurses, working in a chronic follow up ward participated in the data collection process. The questionnaire, after being translated into Amharic by a language expert, was retranslated back into English by another expert for the consistency of the questionnaire. A pretest was conducted in Tibebe Ghion Specialized Hospital, and then the questionnaire was amended accordingly.

The data collectors were supervised by a supervisor and the principal investigator during the data collection period. To prevent coronavirus disease 2019 (COVID-19) transmission, the data collectors collect the data by applying the recommended COVID-19 prevention mechanisms.

## Results

### Sociodemographic characteristics of study participants

A total of 428 hypertensive patients were participated in this study, and 99.5% (426) had respond to the survey. The majority of the study participants were 286 (67.1%) male sex, 349 (81.9%) married, 345 (81%) urban dwellers, and 165 (38.7%) had attained primary school (Table 1). The mean (± SD) age and body mass index of the study participants were (52.5 ± 16.8) years and (21.9 ± 2.7) kg/m^2^, respectively.

**Table 1:** Socio-demographic characteristics of study participants and associated factors among hypertensive patients in Gondar Comphrensive and Specialized and Felege Hiwot Referral Hospitals, 2020.

Variables	Categories	Number (%)
Age (year)	< 55	253 (59.4)
	≥ 55	173 (40.6)
Sex	Male	286 (67.1)
	Female	140 (32.9)
Religion	Orthodox	318 (74.7)
	Muslim	76 (17.8)
	Protestant	32 (7.5)
Occupation	Employed	241 (57.5)
	Merchant	60 (14.5)
	Farmer	71 (15.3)
	Housewife	54 (12.7)
Educational level	Unable to read and write	79 (18.5)
	≤ 8 grade	165 (38.7)
	Grade 9–12	93 (21.3)
	College and above	89 (20.9)
Marital status	Single	50 (11.7)
	Married	342 (80.3)
	Divorced	14 (3.3)
	Widowed	20 (4.7)
Income (ETB)	≤ 1500	133 (31.2)
	1501–3500	102 (23.9)
	≥ 3501	191 (44.8)
Residence	Urban	345 (81)
	Rural	81 (19)
BMI (kg/m^2^)	Normal	301 (70.7)
	Underweight	39 (9.1)
	Overweight	86 (20.2)

*BMI* body mass index, *ETB* Ethiopia Birr.

### Clinical factors of the study participants

Most of the patients, 271 (64%) had been living with the disease for less than 5 years and had a mean creatinine of 1.1 mg/dl. The majority of the patients had 257 (60.3%) controlled blood pressure. The mean (± SD) systolic, and diastolic blood pressure of study participants were 119 ± 10.9 mg/dl, and 74.9 ± 7.7 mmHg, respectively (Table 2).

**Table 2:** Clinical characteristics of study participants among hypertensive patients at the University of Gondar Comprehensive Specialized and Felege Hiwot Referral Hospitals.

Variables	Categories	Number (%)
Creatinine(mg/dl) (mean ± SD)	1.096	1.096 ± 0.516
Anemia	Yes	75 (17.6)
	No	351 (82.4)
Disease duration (year)	< 5	271 (63.6)
	≥ 5	155 (36.4)
Blood pressure status (mmHg)	Controlled	257 (60.3)
	Uncontrolled	169 (39.7)

### Prevalence of anemia among hypertensive patients

**Figure 2:**
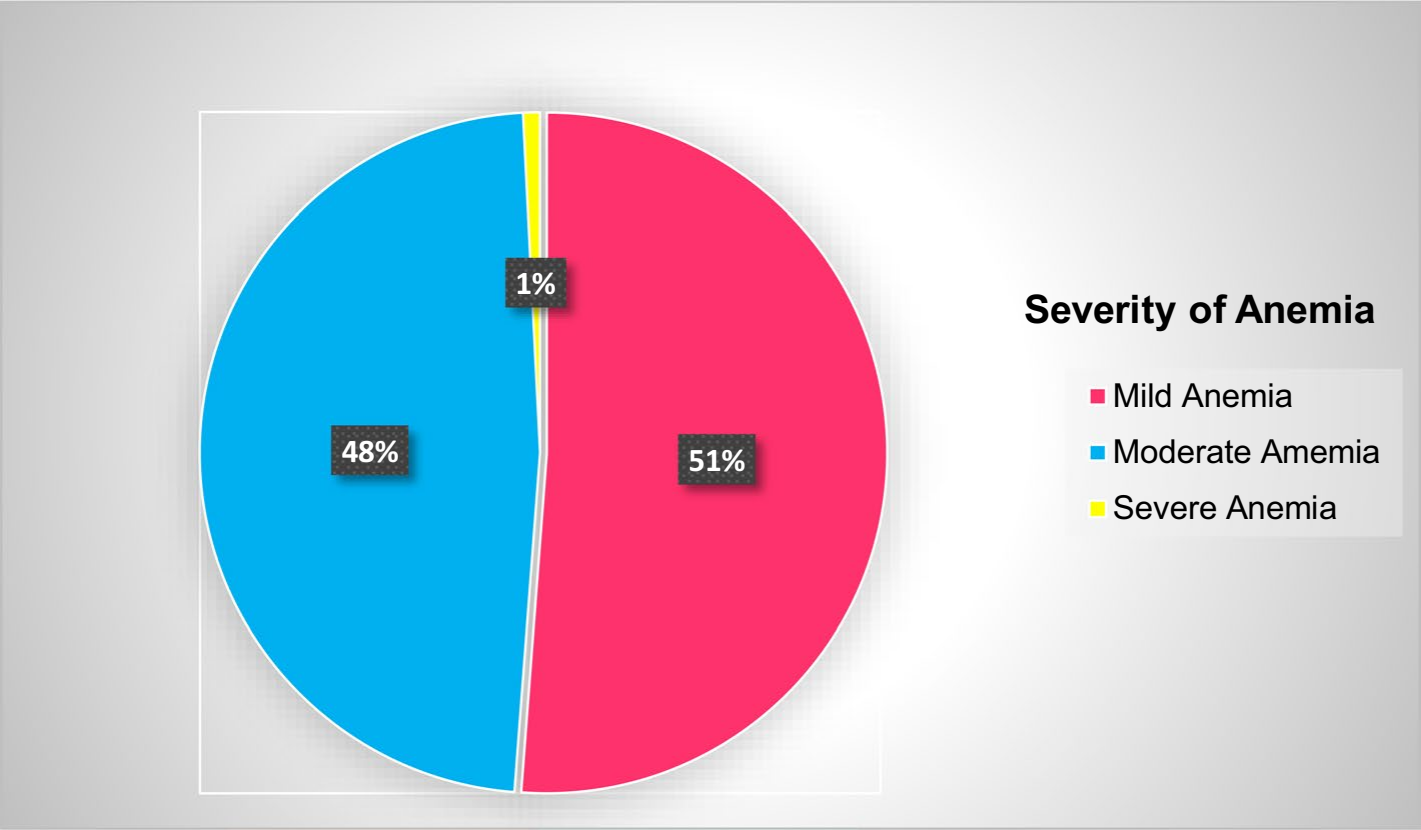
Prevalence of anemia among hypertensive patients at the University of Gondar Comprehensive Specialized and Felege Hiwot Referral Hospitals in 2020.

The mean hemoglobin level of the study participants was 12.7 gm/dl (SD ± 1.5). The prevalence of anemia was 17.6%, with 95% CI (14.3%-21.5%) among hypertensive patients. Among anemic hypertensive patients, the majority of the hypertensive patients had mild anemia 64 (51.2%) (Fig. 2).

### Factors associated with anemia among hypertensive patients

In bivariable logistic regression, estimated glomerular filtration, age, income level, marital status, duration of the disease, educational level, blood pressure status, and pulse pressure were variables associated with anemia at p value < 0.25.

In the multivariable logistic regression, estimated glomerular filtration rate, duration of the disease, blood pressure status, and pulse pressure were variables associated with anemia.

Those patients with uncontrolled blood pressure were 1.91 (AOR = 1.91, 95% CI 1.08–3.35) times more likely to develop anemia as compared to those patients who had controlled blood pressure.

A one-unit increase in the pulse pressure of study participants led to a 1.05 fold (AOR = 1.05 (95% CI 1.02–1.08) increase in the odds of developing anemia.

The odds of having anemia was 2.37 (AOR = 2.37, 95% CI 1.36–4.15) times higher for those patients who had been living with the disease for ≥ 5 years as compared to less than 5 years.

Hypertensive patients with eGFR < 90 ml/min were 2.77 times (AOR = 2.77, 95% CI 1.56–4.92)] more likely to develop anemia than those patients having eGFR ≥ 90 ml/min (Table 3).

**Table 3:** Factors associated with anemia among hypertensive patients at the University of Gondar Comprehensive Specialized and Felege Hiwot Referral Hospitals.

Variables	Categories	Anemia			OR (95% CI)	
		Total (N) %	Yes (N) %	No (N) %	COR	AOR
Age (year)^c^	52.6 ± 16.8	426	75	351	1.02 (1.00–1.03)	1.02 (1.00–1.04)
Blood pressure status	Uncontrolled	257 (64.1)	136 (41.7)	222 (22.4)	1.97 (1.19–3.25)	1.91 (1.08–3.35)*
	Controlled	169 (35.9)	40 (7.7)	129 (28.2)	1	1
eGFR^c^ (ml/min/m^2^)	≥ 90				1	1
	< 90				0.98 (0.97–0.99)	2.77 (1.56- 4.92)*
Disease duration (year)	< 5	271 (62.6)	33 (8.3)	238 (54.3)	1	1
	≥ 5	115 (37.4)	113 (21.8)	42 (15.6)	2.7 (1.61–4.45)	2.37 (1.36–4.15)*
Educational level	College and above	89 (21.2)	14 (9.5)	75 (11.7)	1	1
	Unable to read and write	79 (20.55)	11 (5.5)	68 (15)	0.87 (0.37–2.04)	0.47 (0.17–1.26)
	≤ 8 grade	165 (39.9)	29 (11)	136 (28.8)	1.14 (0.57–2.29)	0.76 (0.34–1.96)
	Grade 9–12	99 (18.4)	21 (4)	21 (14.4)	1.56 (0.4–3.31)	1.71 (0.74–3.93)
Pulse pressure^C^	44 ± 10.2				1.05 (1.02–1.07)	1.05 (1.02–1.08)
Marital status	Single	50 (11.7)	43 (10.1)	7 (1.6)	1	1
	Married	342 (80.3)	286 (66.4)	56 (13.1)	1.20 (0.51–2.81)	1.01 (0.39–2.57)
	Divorced	20 (4.7)	9 (2.1)	5 (2.6)	3.41 (0.88–13.21)	3.24 (0.68–15.36)
	Widowed	14 (3.3)	13(3.1)	7(0.2)	3.31 (0.99–11.17)	3.22 (0.83–12.47)
Income level (ETB)	≥ 3501	191 (44.9)	164 (38.6)	27 (6.3)	1	1
	≤ 1500	133 (31.2)	102 (23.9)	31 (7.3)	1.85 (1.04–3.27)	1.51 (0.79–2.89)
	1501–3500	102 (23.9)	85 (19.9)	17 (4)	1.21 (0.63–2.35)	1.25 (0.60–2.62)

*C* continuous variables, *GFR* estimated glomerular filtration rate, *CI* confidence Interval, *COR* crude odds ratio, *eGFR* estimated glomerular filtration rate, *OR* odds ratio, *N* number.

*p value ≤ 0.05.

## Discussion

Hypertension is a major health problem worldwide and it is associated with an increased risk of cardiovascular disease. Even though hypertension has an impact on red blood cells, which increases the risk of cardiovascular complications, there is a scarcity of data pertaining to the prevalence of anemia among hypertensive patients. Therefore, to fill this gap this study aimed to determine the prevalence of anemia and associated factors among hypertensive patients.

The prevalence of anemia was 17.6% (95% CI 14.3–21.5%) among hypertensive patients. This result was similar to the study conducted in Australia (16%)^4^. But less than a study done in the USA (28%)^29^. This might be due to the sociodemographic difference in which the mean age of our study participants was lower. As age increase, the probability of being anemic increased^30^.

The pathophysiology of anemia in hypertensive patients can be explained in a variety of ways. Patients suffering from essential hypertension showed decreased deformability of red blood cells (RBC)^11, 31^. In hypertensive patients, there is a high intracellular concentration of sodium in red blood cells. This is due to impaired Na–K^+^ ATPase activity in hypertensive patients^11^. Again, hypertensive erythrocytes demonstrate membrane lipid peroxidation and reduced anti-oxidative enzyme activities, which expose the cell membrane to oxidative stress^31^. Therefore, those factors exposed the hypertensive patients’ RBC for hemolytic anemia. Endothelial dysfunction is another common phenomenon in hypertension that promotes the production of pro-inflammatory cytokines, which can lead to the suppression of erythropoiesis and resistance of the bone marrow to erythropoietin stimulation^4, 32, 33^. A high level of circulatory inflammatory cytokines such as tumor necrosis factor and interleukin-6 has a direct inhibitory effect on iron absorption from the gut and iron release from store cells via the hepcidin-mediated pathway, resulting in anemia^34^.

The majority of studies found a link between antihypertensive medication and anemia. Angiotensin-converting enzyme inhibitors and angiotensin II receptor blockers are antihypertensive medications that most commonly affect erythropoiesis process^35^.

Those patients with uncontrolled blood pressure were 1.91 times more likely to develop anemia as compared to those patients who had controlled blood pressure. This finding is in line with the other studies^4, 15, 36, 37^. As blood pressure increases, there is an increment in blood viscosity, the decrease in red blood cell (RBC) deformability, the formation of RBC rouleaux and RBC aggregates, which might result hemolysis of red blood cells^33, 38^.

A one-unit increase in hypertensive patients' pulse pressure (PP) resulted in a 1.1 fold increase in the odds of developing anemia, which is consistent with other studies^15, 39^. Studies showed, a high level of PP (> 80 mmHg) is strongly associated with arterial stiffness and can act as an independent risk factor for cardiovascular complications. This again suggests the necessity of close monitoring of anemia treatment^39^. Cardiovascular mortality significantly and regularly increases with increasing PP^40^. The mechanism for the relationship between anemia and PP remains unclear. However, there are previous studies that shows anemia was positively associated with a high PP. It associated positively with systolic blood pressure (SBP), but was inversely associated with diastolic blood pressure (DBP). Anemia also positively associated with arterial stiffness^15, 41^. Arterial stiffness is common presentation as pulse pressure increase^15^. One of the reasons is that SBP increased with a decrease in hemoglobin and hemoglobin but DBP decreased with a reduction in hemoglobin and hematocrits^15, 33^.

The odds of having anemia was 2.37 times more for those patients who had been living with the disease ≥ 5 years as compared to less than 5 years, in line with another study^33, 42^. Hypertensive patients with eGFR < 90 ml/min were 2.77 times more likely to develop anemia than those patients having eGFR ≥ 90 ml/min. Impaired glomerular filtration rate might be associated to anemia due to decreasing erythropoietin synthesis by the kidney. Studies showed that in essential hypertension there is mild renal insufficiency which might be linked to anemia^43, 44^. This renal impairment leads to anemia though compromised production of erythropoietin by peritubular fibroblast of the kidney^45^. As glomerular filtration rate decrease, renin angiotensin aldosterone system activated which increases the blood volume thereby causes haemodilution anemia in hypertensive patients^33^.

Low hemoglobin levels are associated with increased morbidity and mortality in hypertensive patients^4, 46^. In general, hypertensive patients' microcirculation is worsened and hypertensive complications are increased due to decreased red cell deformability and red blood cell membrane damage. The findings of this study are useful for better treatment with respect to minimizing the risk of complications developing in hypertensive patients.

As a result, hypertensive patients should have their hemoglobin levels checked on a regular basis to avoid end-organ damage.

### Limitation of the study

In this investigation, glomerular filtration rate could be influenced by temporary changes in creatinine levels caused by a variety of variables that cannot be ruled out. Because only a single serum creatinine level in the medical record was used to compute the patients' eGFR. Since secondary data was used for the levels of hemoglobin and creatinine, it might affect the reliability of the data. The morphology of red blood cells was not included in this study, which is recommended for other researchers to study with a stronger study design to explore the cause-and-effect relationship between anemia and hypertension.

## Conclusions

Nearly one out of five hypertensive patients had anemia. Most of the patients had mild anemia. Impaired estimated glomerular filtration rate, duration of HTN, blood pressure status, and pulse pressure were the independent predictors of anemia among hypertensive patients.

It is recommended that hypertensive patients have their hemoglobin levels checked at regular intervals in order to receive appropriate treatment and prevent hypertension-related complications.

## Data Availability

The data will be available upon request from the corresponding author.

## Abbreviations

DBP: Diastolic blood pressure
eGFR: Estimated glomerular filtration rate
EPI: Epidemiology collaboration
HTN: Hypertension
PP: Pulse pressure
NCDs: Non-communicable diseases
RBC: Red blood cell
SBP: Systolic blood pressure
